# Supplementary material for: Resolving the full spectrum of human genome variation using Linked-Reads
Source: Genome Res. 2019 Apr;29(4):635–45. doi: 10.1101/gr.234443.118 (PMC6442396; doi:10.1101/gr.234443.118)
Supplement: Supplemental Material [file supp_gr.234443.118_Supplemental_longranger-2.2.zip › longranger-2.2.tar.bz2!/lr2.2-public/lib/go/src/code.google.com/vitess/docs/user-guide/vschema.html]

Vitess / VSchema User Guide


Toggle navigation

- Overview
- Guides
- Reference
- Blog
- GitHub

- #### Overview

  - What is Vitess
  - Scaling MySQL with Vitess
  - Key Concepts
- #### Getting Started

  - Run Vitess on Kubernetes
    - Custom Docker Build
  - Run Vitess Locally
- #### User Guide

  - Introduction- Client Libraries- Backing Up Data- Reparenting
        - Schema Management
        - VSchema Guide
        - Schema Swap (Tutorial)
        - Sharding
          - Horizontal Sharding (Tutorial, automated)
          - Horizontal Sharding (Tutorial, manual)
          - Sharding in Kubernetes (Tutorial, automated)
          - Sharding in Kubernetes (Tutorial, manual)
        - Vitess Sequences
        - MySQL Server Protocol
        - Vitess and Replication
        - Update Stream
        - Row Based Replication
        - Topology Service
        - Transport Security Model
        - Launching
          - Scalability Philosophy
          - Production Planning
          - Server Configuration
          - 2PC Guide
          - Troubleshooting
        - Upgrading
- #### Reference Guides

  - Vitess API- vtctl Commands
- #### Other Resources

  - Presentations- Blog- Roadmap
- #### Contributing

  - Contributing to Vitess- GitHub Workflow- Code Reviews
- GitHub

# VSchema User Guide

- #### Overview

  - What is Vitess
  - Scaling MySQL with Vitess
  - Key Concepts
- #### Getting Started

  - Run Vitess on Kubernetes
    - Custom Docker Build
  - Run Vitess Locally
- #### User Guide

  - Introduction- Client Libraries- Backing Up Data- Reparenting
        - Schema Management
        - VSchema Guide
        - Schema Swap (Tutorial)
        - Sharding
          - Horizontal Sharding (Tutorial, automated)
          - Horizontal Sharding (Tutorial, manual)
          - Sharding in Kubernetes (Tutorial, automated)
          - Sharding in Kubernetes (Tutorial, manual)
        - Vitess Sequences
        - MySQL Server Protocol
        - Vitess and Replication
        - Update Stream
        - Row Based Replication
        - Topology Service
        - Transport Security Model
        - Launching
          - Scalability Philosophy
          - Production Planning
          - Server Configuration
          - 2PC Guide
          - Troubleshooting
        - Upgrading
- #### Reference Guides

  - Vitess API- vtctl Commands
- #### Other Resources

  - Presentations- Blog- Roadmap
- #### Contributing

  - Contributing to Vitess- GitHub Workflow- Code Reviews

# VSchema User Guide

VSchema stands for Vitess Schema. In contrast to a traditional database schema that contains metadata about tables, a VSchema contains metadata about how tables are organized across keyspaces and shards. Simply put, it contains the information needed to make Vitess look like a single database server.

For example, the VSchema will contain the information about the sharding key for a sharded table. When the application issues a query with a WHERE clause that references the key, the VSchema information will be used to route the query to the appropriate shard.

## Sharding Model

In Vitess, a `keyspace` is sharded by `keyspace ID` ranges. Each row is assigned a keyspace ID, which acts like a street address, and it determines the shard where the row lives. In some respect, one could say that the `keyspace ID` is the equivalent of a NoSQL sharding key. However, there are some differences:

1. The `keyspace ID` is a concept that is internal to Vitess. The application does not need to know anything about it.
2. There is no physical column that stores the actual `keyspace ID`. This value is computed as needed.

This difference is significant enough that we do not refer to the keyspace ID as the sharding key. we will later introduce the concept of a Primary Vindex which more closely ressembles the NoSQL sharding key.

Mapping to a `keyspace ID`, and then to a shard, gives us the flexibility to reshard the data with minimal disruption because the `keyspace ID` of each row remains unchanged through the process.

## Vindex

The Sharding Key is a concept that was introduced by NoSQL datastores. It is based on the fact that there is only one access path to the data, which is the Key. However, relational databases are more versatile about the data and their relationships. So, sharding a database by only designating a sharding key is often insufficient.

If one were to draw an analogy, the indexes in a database would be the equivalent of the key in a NoSQL datastore, except that databases allow you to define multiple indexes per table, and there are many types of indexes. Extending this analogy to a sharded database results in different types of cross-shard indexes. In Vitess, these are called Vindexes.

Simplistically stated, a Vindex provides a way to map a column value to a `keyspace ID`. This mapping can be used to identify the location of a row. A variety of vindexes are available to choose from with different trade-offs, and you can choose one that best suits your needs.

Vindexes offer many flexibilities:

- A table can have multiple Vindexes.
- Vindexes could be NonUnique, which allows a column value to yield multiple keyspace IDs.
- They could be a simple function or be based on a lookup table.
- They could be shared across multiple tables.
- Custom Vindexes can be plugged in, and Vitess will still know how to reshard using such Vindexes.

### The Primary Vindex

The Primary Vindex is analogous to a database primary key. Every sharded table must have one defined. A Primary Vindex must be unique: given an input value, it must produce a single keyspace ID. This unique mapping will be used at the time of insert to decide the target shard for a row. Conceptually, this is also equivalent to the NoSQL Sharding Key, and we often refer to the Primary Vindex as the Sharding Key.

However, there is a subtle difference: NoSQL datastores let you choose the Sharding Key, but the Sharding Scheme is generally hardcoded in the engine. In Vitess, the choice of Vindex lets you control how a column value maps to a keyspace ID. In other words, a Primary Vindex in Vitess not only defines the Sharding Key, it also decides the Sharding Scheme.

Vindexes come in many varieties. Some of them can be used as Primary Vindex, and others have different purposes. The following sections will describe their properties.

### Secondary Vindexes

Secondary Vindexes are additional vindexes you can define against other columns of a table offering you optimizations for WHERE clauses that do not use the Primary Vindex. Secondary Vindexes return a single or a limited set of `keyspace IDs` which will allow VTGate to only target shards where the relevant data is present. In the absence of a Secondary Vindex, VTGate would have to send the query to all shards.

Secondary Vindexes are also commonly known as cross-shard indexes. It is important to note that Secondary Vindexes are only for making routing decisions. The underlying database shards will most likely need traditional indexes on those same columns.

### Unique and NonUnique Vindex

A Unique Vindex is one that yields at most one keyspace ID for a given input. Knowing that a Vindex is Unique is useful because VTGate can push down some complex queries into VTTablet if it knows that the scope of that query cannot exceed a shard. Uniqueness is also a prerequisite for a Vindex to be used as Primary Vindex.

A NonUnique Vindex is analogous to a database non-unique index. It is a secondary index for searching by an alternate WHERE clause. An input value could yield multiple keyspace IDs, and rows could be matched from multiple shards. For example, if a table has a `name` column that allows duplicates, you can define a cross-shard NonUnique Vindex for it, and this will let you efficiently search for users that match a certain `name`.

### Functional and Lookup Vindex

A Functional Vindex is one where the column value to keyspace ID mapping is pre-established, typically through an algorithmic function. In contrast, a Lookup Vindex is one that gives you the ability to create an association between a value and a keyspace ID, and recall it later when needed.

Typically, the Primary Vindex is Functional. In some cases, it is the identity function where the input value yields itself as the kesypace id. However, one could also choose other algorithms like hashing or mod functions.

A Lookup Vindex is usually backed by a lookup table. This is analogous to the traditional database index, except that it is cross-shard. At the time of insert, the computed keyspace ID of the row is stored in the lookup table against the column value.

### Shared Vindexes

Relational databases encourage normalization, which lets you split data into different tables to avoid duplication in the case of one-to-many relationships. In such cases, a key is shared between the two tables to indicate that the rows are related, aka `Foreign Key`.

In a sharded environment, it is often beneficial to keep those rows in the same shard. If a Lookup Vindex was created on the foreign key column of each of those tables, you would find that the backing tables would actually be identical. In such cases, Vitess lets you share a single Lookup Vindex for multiple tables. Of these, one of them is designated as the owner, which is responsible for creating and deleting these associations. The other tables just reuse these associations.

Caveat: If you delete a row from the owner table, Vitess will not perform cascading deletes. This is mainly for efficiency reasons; The application is likely capable of doing this more efficiently.

Functional Vindexes can be also be shared. However, there is no concept of ownership because the column to keyspace ID mapping is pre-established.

### Orthogonality

The previously described properties are mostly orthogonal. Combining them gives rise to the following valid categories:

- **Functional Unique**: This is the most popular category because it is the one best suited to be a Primary Vindex.
- **Functional NonUnique**: There are currently no use cases that need this category.
- **Lookup Unique Owned**: This gets used for optimizing high QPS queries that do not use the Primary Vindex columns in their WHERE clause. There is a price to pay: You incur an extra write to the lookup table for insert and delete operations, and an extra lookup for read operations. However, it is worth it if you do not want these high QPS queries to be sent to all shards.
- **Lookup Unique Unowned**: This category is used as an optimization as described in the Shared Vindexes section.
- **Lookup NonUnique Owned**: This gets used for high QPS queries on columns that are non-unique.
- **Lookup NonUnique Unowned**: You would rarely have to use this category because it is unlikely that you will be using a column as foreign key that is not unique within a shard. But it is theoretically possible.

Of the above categories, `Functional Unique` and `Lookup Unique Unowned` Vindexes can be Primary. This is because those are the only ones that are unique and have the column to keyspace ID mapping pre-established. This is required because the Primary Vindex is responsible for assigning the keyspace ID for a row when it is created.

However, it is generally not recommended to use a Lookup vindex as Primary because it is too slow for resharding. If absolutely unavoidable, you can use a Lookup Vindex as Primary. In such cases, it is recommended that you add a `keyspace ID` column to such tables. While resharding, Vitess can use that column to efficiently compute the target shard. You can even configure Vitess to auto-populate that column on inserts. This is done using the reverse map feature explained below.

### How vindexes are used

#### Cost

Vindexes have costs. For routing a query, the Vindex with the lowest cost is chosen. The current costs are:

| Vindex Type | Cost |
| --- | --- |
| Indentity | 0 |
| Functional | 1 |
| Lookup Unique | 10 |
| Lookup NonUnique | 20 |

#### Select

In the case of a simple select, Vitess scans the WHERE clause to match references to Vindex columns and chooses the best one to use. If there is no match and the query is simple without complex constructs like aggreates, etc, it is sent to all shards.

Vitess can handle more complex queries. For now, you can refer to the design doc on how it handles them.

#### Insert

- The Primary Vindex is used to generate a keyspace ID.
- The keyspace ID is validated against the rest of the Vindexes on the table. There must exist a mapping from the column value to the keyspace ID.
- If a column value was not provided for a Vindex and the Vindex is capable of reverse mapping a keyspace ID to an input value, that function is used to auto-fill the column. If there is no reverse map, it is an error.

#### Update

The WHERE clause is used to route the update. Changing the value of a Vindex column is unsupported because this may result in a row being migrated from one shard to another.

#### Delete

If the table owns lookup vindexes, then the rows to be deleted are first read and the associated Vindex entries are deleted. Following this, the query is routed according to the WHERE clause.

### Predefined Vindexes

Vitess provides the following predefined Vindexes:

| Name | Type | Description | Primary | Reversible | Cost |
| --- | --- | --- | --- | --- | --- |
| binary | Functional Unique | Identity | Yes | Yes | 0 |
| binary\_md5 | Functional Unique | md5 hash | Yes | No | 1 |
| hash | Functional Unique | 3DES null-key hash | Yes | Yes | 1 |
| lookup | Lookup NonUnique | Lookup table non-unique values | No | Yes | 20 |
| lookup\_unique | Lookup Unique | Lookup table unique values | If unowned | Yes | 10 |
| numeric | Functional Unique | Identity | Yes | Yes | 0 |
| numeric\_static\_map | Functional Unique | A JSON file that maps input values to keyspace IDs | Yes | No | 1 |
| unicode\_loose\_md5 | Functional Unique | Case-insensitive (UCA level 1) md5 hash | Yes | No | 1 |

Custom vindexes can also be plugged in as needed.

## Sequences

Auto-increment columns do not work very well for sharded tables. Vitess sequences solve this problem. Sequence tables must be specified in the VSchema, and then tied to table columns. At the time of insert, if no value is specified for such a column, VTGate will generate a number for it using the sequence table.

## VSchema

As mentioned in the beginning of the document, a VSchema is needed to tie together all the databases that Vitess manages. For a very trivial setup where there is only one unsharded keyspace, there is no need to specify a VSchema because Vitess will know that there is no other place to route a query.

If you have multiple unsharded keyspaces, you can still avoid defining a VSchema in one of two ways:

1. Connect to a keyspace and all queries are sent to it.
2. Connect to Vitess without specifying a keyspace, but use qualifed names for tables, like `keyspace.table` in your queries.

However, once the setup exceeds the above complexity, VSchemas become a necessity. Vitess has a working demo of VSchemas. This section documents the various features highlighted with snippets pulled from the demo.

### Unsharded Table

The following snippets show the necessary configs for creating a table in an unsharded keyspace:

Schema:

```
# lookup keyspace
create table name_user_idx(name varchar(128), user_id bigint, primary key(name, user_id));
```

VSchema:

```
// lookup keyspace
{
  "sharded": false,
  "tables": {
    "name_user_idx": {}
  }
}
```

For a normal unsharded table, the VSchema only needs to know the table name. No additional metadata is needed.

### Sharded Table With Simple Primary Vindex

To create a sharded table with a simple Primary Vindex, the VSchema requires more information:

Schema:

```
# user keyspace
create table user(user_id bigint, name varchar(128), primary key(user_id));
```

VSchema:

```
// user keyspace
{
  "sharded": true,
  "vindexes": {
    "hash": {
      "type": "hash"
    },
  "tables": {
    "user": {
      "column_vindexes": [
        {
          "column": "user_id",
          "name": "hash"
        }
      ]
    }
  }
}
```

Because Vindexes can be shared, the JSON requires them to be specified in a separate `vindexes` section, and then referenced by name from the `tables` section. The VSchema above simply states that `user_id` uses `hash` as Primary Vindex. The first Vindex of every table must be the Primary Vindex.

### Specifying A Sequence

Since user is a sharded table, it will be beneficial to tie it to a Sequence. However, the sequence must be defined in the lookup (unsharded) keyspace. It is then referred from the user (sharded) keyspace. In this example, we are designating the user\_id (Primary Vindex) column as the auto-increment.

Schema:

```
# lookup keyspace
create table user_seq(id int, next_id bigint, cache bigint, primary key(id)) comment 'vitess_sequence';
insert into user_seq(id, next_id, cache) values(0, 1, 3);
```

For the sequence table, `id` is always 0. `next_id` starts off as 1, and the cache is usually a medium-sized number like 1000. In our example, we are using a small number to showcase how it works.

VSchema:

```
// lookup keyspace
{
  "sharded": false,
  "tables": {
    "user_seq": {
      "type": "sequence"
    }
  }
}

// user keyspace
{
  "sharded": true,
  "vindexes": {
    "hash": {
      "type": "hash"
    },
  "tables": {
    "user": {
      "column_vindexes": [
        {
          "column": "user_id",
          "name": "hash"
        }
      ],
      "auto_increment": {
        "column": "user_id",
        "sequence": "user_seq"
      }
    }
  }
}
```

### Specifying A Secondary Vindex

The following snippet shows how to configure a Secondary Vindex that is backed by a lookup table. In this case, the lookup table is configured to be in the unsharded lookup keyspace:

Schema:

```
# lookup keyspace
create table name_user_idx(name varchar(128), user_id bigint, primary key(name, user_id));
```

VSchema:

```
// lookup keyspace
{
  "sharded": false,
  "tables": {
    "name_user_idx": {}
  }
}

// user keyspace
{
  "sharded": true,
  "vindexes": {
    "name_user_idx": {
      "type": "lookup_hash",
      "params": {
        "table": "name_user_idx",
        "from": "name",
        "to": "user_id"
      },
      "owner": "user"
    },
  "tables": {
    "user": {
      "column_vindexes": [
        {
          "column": "name",
          "name": "name_user_idx"
        }
      ]
    }
  }
}
```

To recap, a checklist for creating the shared Secondary Vindex is:

- Create physical `name_user_idx` table in lookup database.
- Define a routing for it in the lookup VSchema.
- Define a Vindex as type `lookup_hash` that points to it. Ensure that the `params` match the table name and columns.
- Define the owner for the Vindex as the `user` table.
- Specify that `name` uses the Vindex.

Currently, these steps have to be currently performed manually. However, extended DDLs backed by improved automation will simplify these tasks in the future.

### Advanced usage

The examples/demo also shows more tricks you can perform:

- The `music` table uses a secondary lookup vindex `music_user_idx`. However, this lookup vindex is itself a sharded table.
- `music_extra` shares `music_user_idx` with `music`, and uses it as Primary Vindex.
- `music_extra` defines an additional Functional Vindex called `keyspace_id` which the demo auto-populates using the reverse mapping capability.
- There is also a `name_info` table that showcases a case-insensitive Vindex `unicode_loose_md5`.

## Roadmap

VSchema is still evolving. Features are mostly added on demand. The following features are currently on our roadmap:

- DDL support
- Lookup Vindex backfill
- Pinned tables: This feature will allow unsharded tables to be pinned to a keypsace id. This avoids the need for a separate unsharded keyspace to contain them.

Contact: vitess@googlegroups.com  **·**  
Announcements  **·**  
© 2017 Vitess powered by Google Inc
